# Dehydration of Niclosamide Monohydrate Polymorphs: Different Mechanistic Pathways to the Same Product

**DOI:** 10.1021/acs.cgd.3c00322

**Published:** 2023-06-02

**Authors:** Jen E. Mann, Renee Gao, Jennifer A. Swift

**Affiliations:** † Georgetown University, Department of Chemistry, 37th and O Streets NW, Washington, District of Columbia 20057-1227, United States

## Abstract

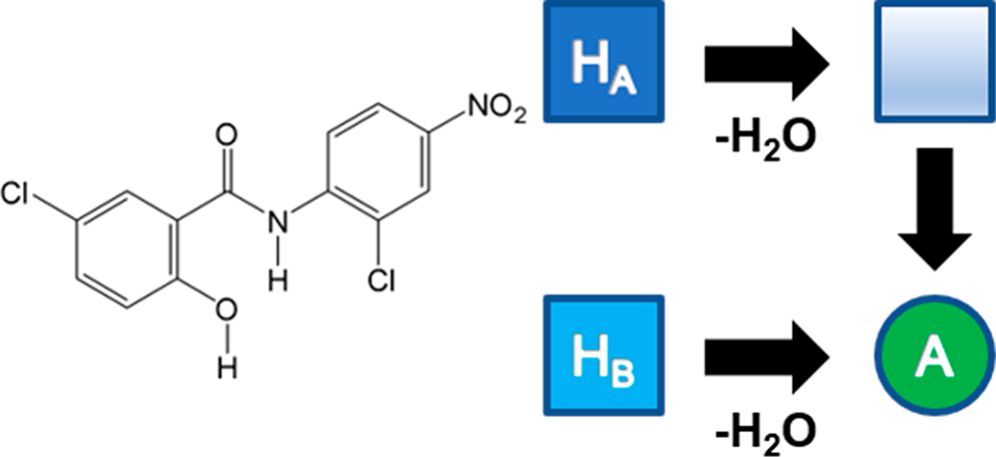

Many active pharmaceutical ingredients (APIs) can crystallize as hydrates or anhydrates, the relative stability of which depends on their internal structures as well as the external environment. Hydrates may dehydrate unexpectedly or intentionally, though the molecular-level mechanisms by which such transformations occur are difficult to predict *a priori*. Niclosamide is an anthelmintic drug on the World Health Organization’s “List of Essential Medicines” that crystallizes in two monohydrate forms: H_A_ and H_B_. Through complementary time-resolved synchrotron powder X-ray diffraction and thermogravimetric kinetic studies, we demonstrate that the two monohydrates dehydrate via distinctly different solid state pathways yet yield the same final anhydrate phase. Water loss from H_A_ via diffusion yields an isomorphous desolvate intermediate which can rearrange to at least two different polymorphs, only one of which exhibits long-term stability. In contrast, dehydration of H_B_ proceeds via a surface nucleation process where simultaneous water loss and product formation occur with no detectable crystalline intermediates. Comparative analysis of the two systems serves to highlight the complex relationship between lattice structure and solid state dehydration processes.

## Abstract 2

The dehydration of two niclosamide monohydrates follows different mechanistic pathways. Through a combination of time-resolved synchrotron powder X-ray diffraction and thermogravimetric studies, water loss and structure rearrangement proceed stepwise in one hydrate and concurrently in the other. Comparison of the two hydrates highlights the complex relationship between lattice structure and solid state dehydration processes.

## Introduction

Many small molecule active pharmaceutical ingredients (APIs) can exist in multiple solid-state forms (e.g., polymorphs, hydrates, solvates, salts, and amorphous phases)^1−3^ which exhibit different physical properties. Solid form screening is an important step in the development of orally administered pharmaceuticals.^4,5^ Hydrates require special consideration, since their relative stabilities with respect to water-free forms depends not only on their internal structure but on the environmental conditions to which they are exposed. Unanticipated conversion to less hydrated forms during manufacturing or under long-term storage can create both product efficacy concerns and intellectual property issues. On the other hand, there is growing recognition that hydrates and other solvates can serve as unique precursors, from which process-induced desolvation may yield novel solvent-free forms that may not be obtainable by conventional crystallization processes.^6−11^

**Figure 1:**
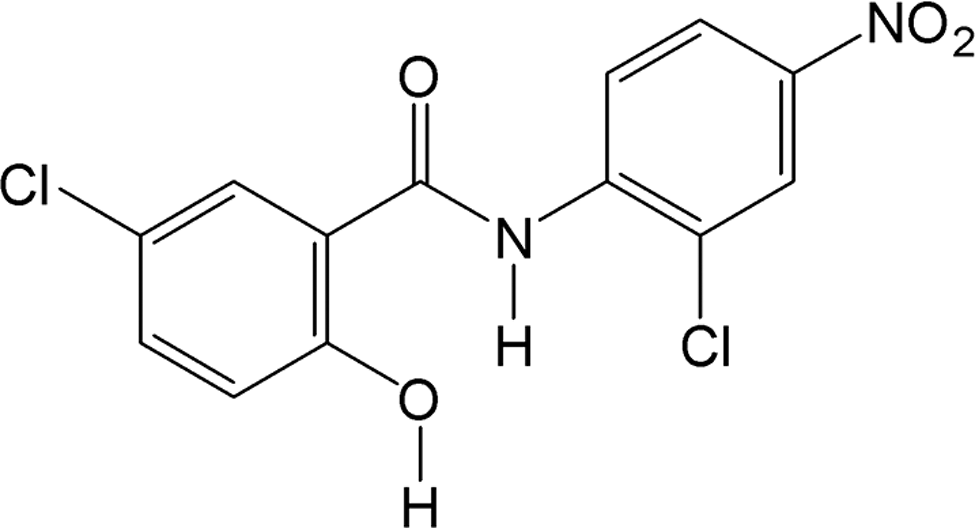
Molecular structure of niclosamide (NCL).

Comparative studies of different API hydrates can provide valuable insight into how structural factors influence the kinetics, mechanisms and products of solid state dehydration reactions. Niclosamide, *N*-(2′-chloro-4′-nitrophenyl)-5-chlorosalicylamide (NCL, Figure 1), is an anthelmintic drug on the World Health Organization’s “List of Essential Medicines”^12^ for its efficacy in treating tapeworm infections. First developed by Bayer,^13^ it was in use as early as 1961 and gained FDA approval in 1982. Several more recent studies have shown that niclosamide exhibits multifunctional activity. Drug repurposing screens have investigated its potential in treating a range of human diseases^14^ including various types of cancer,^15−17^ metabolic disorders,^18^ tuberculosis,^19,20^ and viruses^21−23^ including COVID-19.^24,25^

NCL is known to adopt multiple solid forms, including two solvent-free (single component) polymorphs,^26,27^ two monohydrates,^28,29^ multiple solvates,^28−30^ as well as cocrystals,^27,31^ cocrystal salts,^32^ and an amorphous solid dispersion.^33^ In the present work, we investigate the solid state dehydration of two NCL monohydrates (H_A_ and H_B_) and show that under identical process-induced conditions the two follow entirely different mechanistic pathways to the same final anhydrous product. Using a combination of time-resolved synchrotron powder X-ray diffraction (sPXRD) and thermogravimetry (TGA), the divergent mechanistic pathways are rationalized based on the hydrate lattice topologies and the accessibility of cooperative motions that can facilitate the transformation to the stable anhydrate form.

## Experimental Methods

### Materials

Niclosamide (NCL) was purchased from Sigma-Aldrich (≥98%) and used as received. Ethyl acetate and acetone were obtained from Fisher Scientific and were reagent grade or higher. Water was acquired from a Milli-Q Integral Water Purification System at 18.2 MΩ.

### Preparation of NCL Monohydrates

#### Monohydrate A (H_A_)

H_A_ was obtained by recrystallization from acetone. NCL (150 mg) was added to 50 mL of 18 MΩ ultrapure water and heated with stirring for 15 min. Upon cooling to room temperature, the solid product was vacuum filtered and allowed to dry under ambient conditions. The dry material was placed in 50 mL of acetone, heated in a water bath, and stirred to effect complete dissolution. The growth solution was then transferred to Petri dishes (90 mm diameter) covered with punctured parafilm and maintained at room temperature. Crystals appeared within 5–7 days.

#### Monohydrate B (H_B_)

H_B_ was prepared by recrystallization from ethyl acetate. NCL (150 mg) was dissolved in 50 mL of ethyl acetate through heating in a hot water bath with stirring. The growth solution was then transferred to Petri dishes covered with punctured parafilm and maintained at room temperature. Crystals appeared after 11–14 days.

Images of each hydrated form were collected on an Olympus BX-50 polarizing microscope fitted with a Lumenera Xfinity 2.0 camera.

### Thermal Analysis

The thermal transitions in each of the phases were assessed with differential scanning calorimetry (DSC) and thermogravimetric analysis (TGA). DSC data were collected on a TA Instruments Discovery DSC 25. Samples of phase pure H_A_ or H_B_ (2–5 mg), either as grown and unground or ground with a mortar and pestle, were placed in aluminum pans with unsealed lids. Samples were heated at 5 °C/min from room temperature to 250 °C. The reported thermal transition temperatures are an average of at least triplicate measurements.

Weight loss from each sample was determined using a TA Instruments SDT_Q600 simultaneous TGA-DSC analyzer (New Castle, DE). All samples (2–5 mg) were ground with a mortar and pestle before being placed in open ceramic pans and heated at a rate of 5 °C/min to a maximum temperature of 210 °C. Reported weight losses are an average of at least triplicate measurements. Ground samples of each monohydrate were also heated isothermally at 40, 45, and 50 °C until dehydration was complete. Isothermal experiments at each temperature were performed in triplicate. The calculated water content in the monohydrate is 5.2%.

### Solid State Dehydration Kinetics

Solid state dehydration kinetics were determined from isothermal TGA data at 40, 45, and 50 °C. The fraction dehydrated at each time point (α) was determined from the percent weight loss at each data point relative to the starting experimental mass, as shown in eq 1.
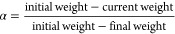
1

Data in the linear regions of the TGA curves (0.1 < α < 0.9 or 0.1 < α < 0.8) were used in established model-based and model-free analyses. In model-based analyses, data were fit to each of the 17 different solid state reaction models in Table 1. All reaction models have the form
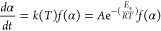
2where the rate constant (*k*) is calculated from the frequency factor (*A*), the activation energy (*E*_a_), the gas constant (*R* = 8.314 J K^–1^ mol^–1^), and the temperature in K (*T*). Each model in differential form is represented as *f*(α) and in general form as *g*(α). The dehydration rate at each temperature was calculated, and the slope of an Arrhenius plot of −ln(*k*) vs 1/*T* was used to calculate the activation energy (*E*_a_) associated with the reaction. The quality of fit for each reaction model was assessed based on the correlation coefficient (*R*^2^). Model-free Friedman and standard analyses^34−37^ were also used to determine the *E*_a_ at select time points throughout the reaction.

**Table 1:** Solid State Reaction Models and Integral Expressions Used for Kinetic Analyses^38−40^

dehydration models	integral equation *g*(α) = *kt*
nucleation and growth	
1D growth of nuclei^a^ (A2)	(−ln(1 – α))^0.5^
2D growth of nuclei^a^ (A3)	(−ln(1 – α))^1/3^
3D growth of nuclei^a^ (A4)	(−ln(1 – α))^1/4^
random nucleation^b^ (B1)	ln(α/(1 – α)) + e^α^
Power law (*n* = 1/2; P2)	α^1/2^
Power law (*n* = 1/3; P3)	α^1/3^
Power law (*n* = 1/4; P4)	α^1/4^
geometrical contraction	
2D phase boundary (R2)	1 – (1 – α)^1/2^
3D phase boundary (R3)	1 – (1 – α)^1/3^
diffusion	
1D diffusion (D1)	α^2^
2D diffusion (D2)	(1 – α)*(ln(1 – α)) + α
3D diffusion^c^ (D3)	(1 – (1 – α)^1/3^)^2^
3D diffusion^d^ (D4)	(1 – (2/3)α) – (1 – α)^2/3^
reaction order	
zero-order (R1)	α
first-order (F1)	–ln(1 – α)
second-order (F2)	(1/(1 – α)) – 1
third-order (F3)	(1/2)*(((1 – α)^−2^) – 1)

a Avrami–Erofeyev equation.

b Prout–Tompkins equation.

c Jander equation.

d Ginstling–Brounshtein equation.

### Powder X-ray Diffraction (PXRD)

PXRD data of ground samples were collected at room temperature on a Rigaku Ultima IV diffractometer (Cu Kα radiation, 40 kV tube voltage, 30 mA current) in order to confirm the identity and phase purity of all materials. Data were collected over 2θ = 3–40° at a scan speed of 2.0°/min and analyzed with X’pert Highscore Plus v2.2 software. Experimental PXRD data were compared against simulated powder patterns from CIF files available in the CSD.^16^ These include H_A_ (refcode: OBEQAN01^26^), H_B_ (OBEQAN^28^), and the two anhydrate polymorphs: form 1 (HEBFUR^27^) and form 2 (HEBFUR01^26^).

### Time-Resolved Synchrotron Powder X-ray Diffraction (sPXRD)

Time-resolved synchrotron powder X-ray diffraction data were collected at the Advanced Photon Source (APS) beamline 17-BM-B. The beamline is equipped with a Si (311) monochromator, a PerkinElmer a-Si Flat Panel PE1621 area detector, and an Oxford Cryosystems Cryostream 700+. Data were collected over three trips with X-ray beam energies of 27.3 keV (λ = 0.45390 Å), 27.4 keV (λ = 0.45256 Å), and 51.5 keV (λ = 0.24087 Å). Variable temperature experiments were performed to a maximum temperature of 210 °C using a heating rate of 6–10 °C/min. In all H_A_ experiments and one H_B_ variable temperature experiment, samples were hand ground in a small amount of growth solution, then loaded into a 1.1 mm Kapton capillary (Cole-Parmer) and stoppered with glass wool at each end. In H_B_ isothermal experiments and one additional variable temperature experiment, samples were hand ground in a minimal amount of growth solution to create a semidry paste, which was then loaded into a 1.1 mm OD quartz capillary and stoppered with glass wool at each end. Capillaries were then mounted in a flow cell,^41^ and *in situ* experiments were performed under a flowing He atmosphere (5 mL/min) with continuous rocking of the sample at 10–15° throughout the experiment. High Q-range sPXRD patterns were collected every ∼20 s by summing over 10 images each with an exposure time of 2.0 s. GSAS-II^42^ software was used to process the images and perform integration. TOPAS-V6^43^ was used for sPXRD pattern refinement.

## Results and Discussion

In any solid state dehydration reaction, there are three general potential outcomes. Upon water loss, the hydrate can (a) retain the same crystal lattice (isomorphous desolvates^44^), (b) transform to a structure with a different lattice, or (c) become amorphous. Dehydration may yield a single anhydrous product or a mixture of phases, and the pathway to the final dehydration product(s) may or may not include other crystalline intermediates. Our incomplete understanding of solid state dehydration mechanisms on the molecular level is due at least in part to the limited experimental methods capable of tracking structural changes on a sufficiently fast time scale. Here, we take advantage of the high resolution and rapid data acquisition times afforded by time-resolved synchrotron powder X-ray diffraction and complementary thermogravimetric kinetic measurements to compare the solid state dehydration of two niclosamide monohydrate polymorphs, H_A_ and H_B_, under the same conditions.

### H_A_ and H_B_ Structures

**Figure 2:**
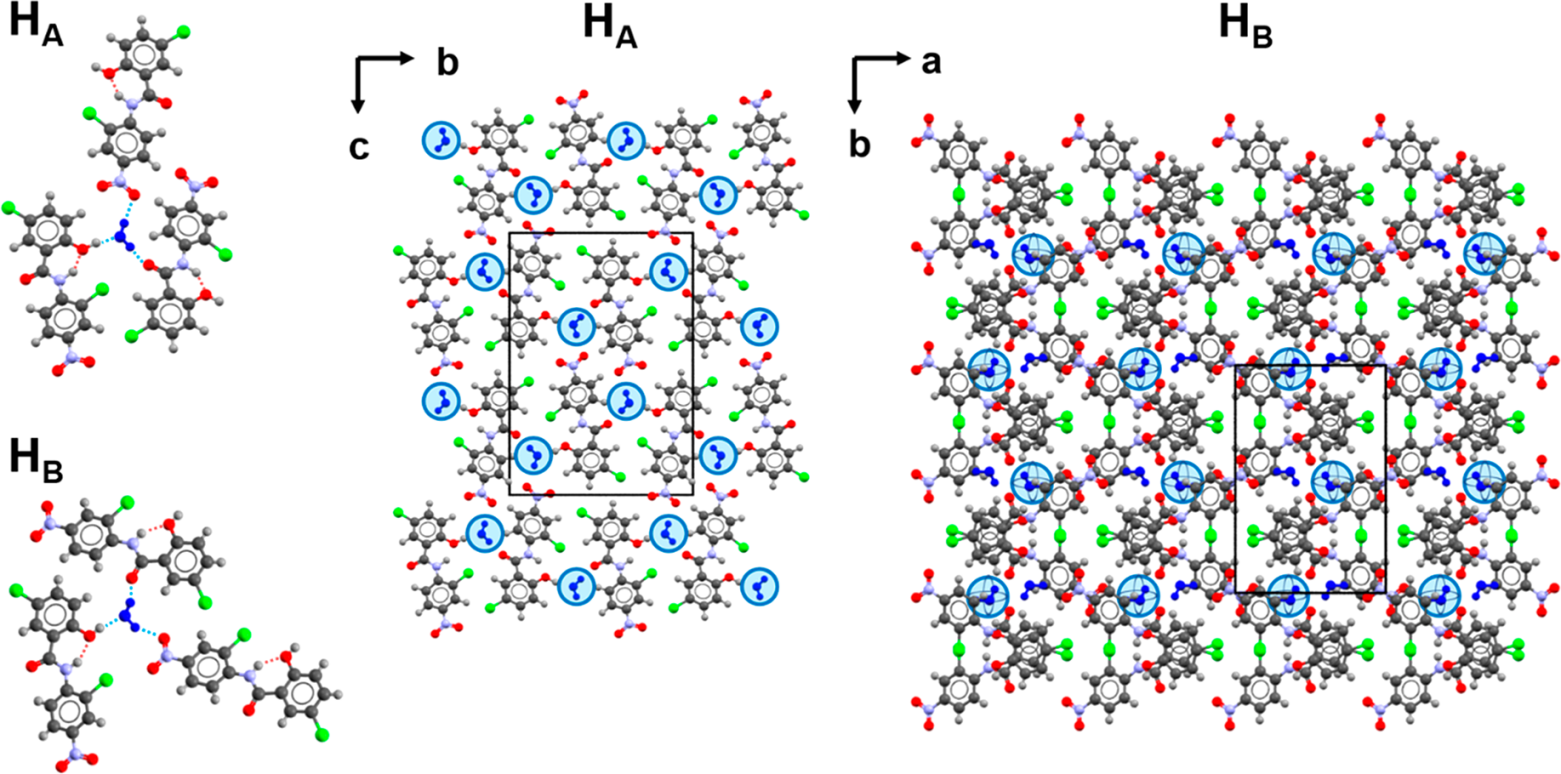
Molecular structures of H_A_ (OBEQAN01) and H_B_ (OBEQAN). Water molecules are colored blue for clarity. (Left) Each water molecule is hydrogen bonded to three NCL molecules shown with blue dotted lines. Intramolecular hydrogen bonds are indicated with orange dotted lines. (Middle) H_A_ viewed down the π-stacking axis (*a* axis). The (100) planes in H_A_ are centrosymmetric, with face-to-face π-stacked molecules related by translation. Water channels are indicated with blue circles. (Right) H_B_ viewed down the π-stacking axis (*c* axis). The (001) planes in H_B_ are polar, with adjacent layers related by a two-fold rotation. This creates face-to-face π stacks of phenols with water molecules in isolated cavities (blue spheres) separated by nitroaromatic rings.

Like all of the NCL forms known to date, NCL molecules in H_A_ and H_B_ adopt an essentially planar conformation which is stabilized by an intramolecular hydrogen bond between the central amide N–H and the oxygen of the chlorophenol ring (graph set S(6)). Water molecules in room temperature structures of both H_A_ (*P*2_1_/*c*: *a* = 3.813 Å, *b* = 16.143 Å, *c* = 23.065 Å, *b* = 92.87°)^26^ and H_B_ (*P*2_1_/*c*: *a* = 7.346 Å, *b* = 11.332 Å, *c* = 16.964 Å, *b* = 98.28°)^28^ hydrogen bond to three neighboring NCL molecules through three different functional groups: (1) a hydroxy group O_W_···H–O, (2) a carbonyl group O_W_–H_W_···O=C, and (3) a nitro group O_W_–H_W_···O(NO) (Figure 2). The O···O distances in H_A_ and H_B_ are similar, with H_A_ distances of 2.66, 2.74, and 3.00 Å, and H_B_ distances of 2.60, 2.85, and 2.93 Å for 1, 2, and 3, respectively.

Despite the similar hydrogen bond types, the water environments in H_A_ and H_B_ differ significantly. Expanded views of the crystal packing make the differences in the local water environment and π-stacking more apparent. In H_A_, (100) layers are nonpolar and related by translation, creating homogeneous face-to-face aromatic π stacks of nitrophenyl rings and phenol rings along the *a* axis (center-to-center distance = 3.81 Å). Between these stacks are one-dimensional channels occupied by water molecules, though neighboring water molecules within a channel are too far apart to bond to one another.

In contrast, the (001) layers in H_B_ are polar, and adjacent layers are related by a 2-fold rotation. This packing motif creates π-stacks of phenol rings along the *c* axis (average center-to-center distance = 3.67 Å) and places water molecules farther away from one another and into isolated cavities separated by nitrophenyl rings.

### Crystal Growth of H_A_ and H_B_

**Figure 3:**
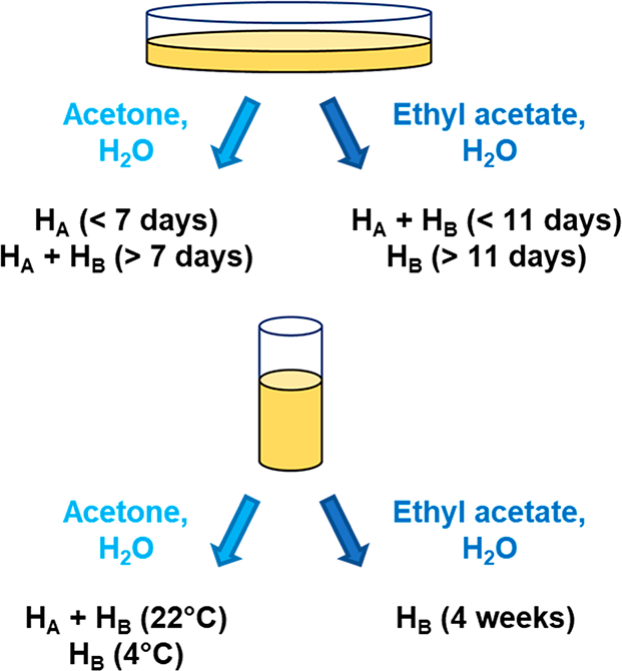
Results of crystal growth from evaporation of wet acetone and ethyl acetate in Petri dishes and glass vials.

Previously reported growth methods yielded monohydrate, though not always in phase pure form. In general, H_A_ was favored from acetone solutions and H_B_ from ethyl acetate, though under some conditions concomitant mixtures of hydrates were obtained (Figure 3). The solution evaporation rate proved to be a significant factor in the outcome. H_A_ could be obtained in phase pure form under fast evaporation conditions from acetone in Petri dishes. Phase pure H_B_ was reliably obtained from ethyl acetate after extended time periods in Petri dishes (≥11 days) or 2 dram vials (∼4 weeks). Phase-pure H_B_ could also be obtained from acetone solutions maintained at 4 °C in 2 dram vials. Both H_A_ and H_B_ adopt needle-like morphologies but can usually be visually distinguished by their different aspect ratios (Figure S1).

### Dehydration of H_A_

**Figure 4:**
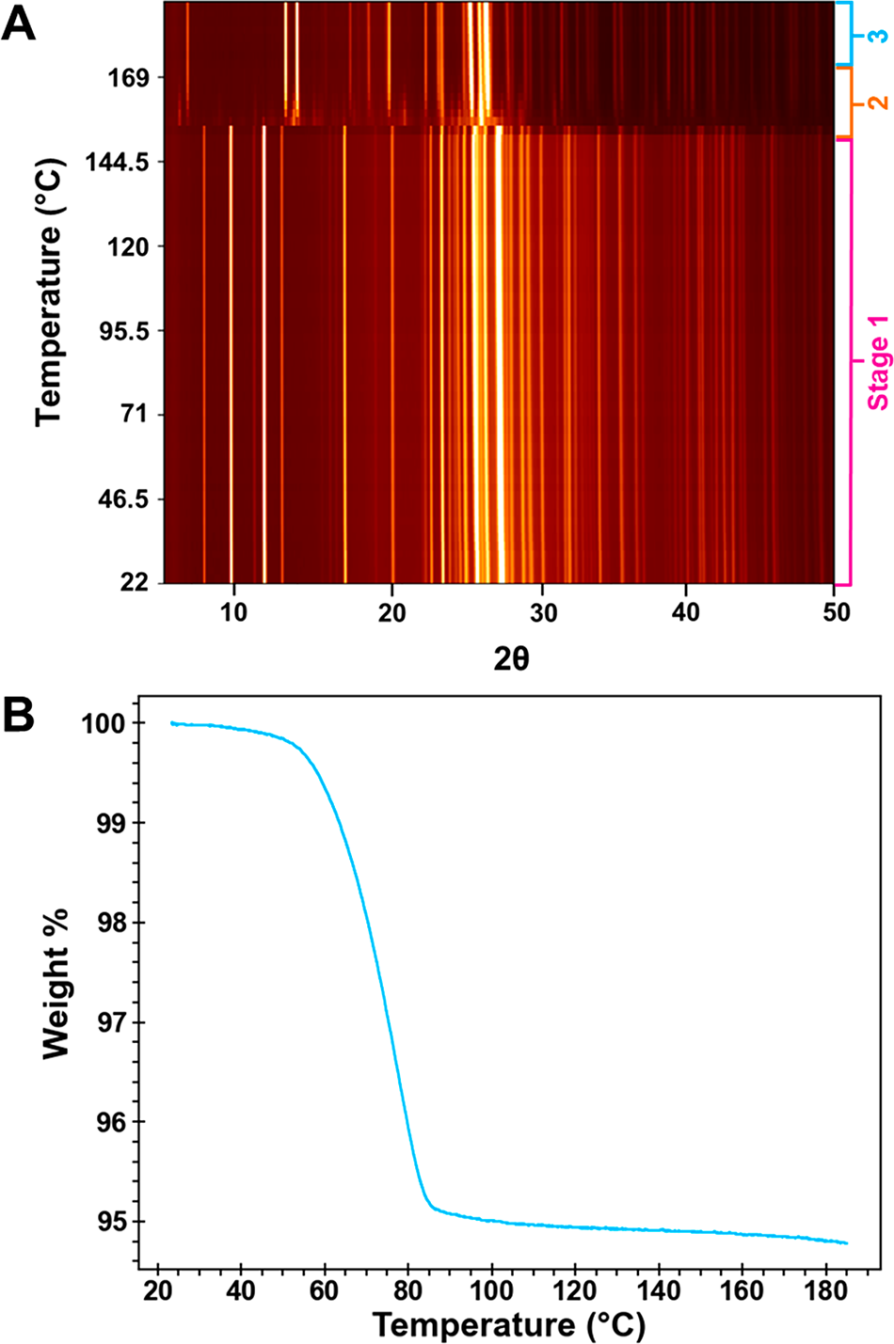
(A) Time-resolved sPXRD of ground H_A_ heated at 6 °C/min from room temperature to 192 °C. The 2θ scale is normalized to Cu Kα. Three different regions are identified as stages 1, 2, and 3. (B) TGA of ground H_A_ heated at 5 °C/min in open pans.

Previous reports on the solid state properties of NCL monohydrates have appeared in the literature.^28,45−50^ Using typical DSC and/or TGA heating conditions, these earlier studies consistently showed that H_A_ dehydrates at temperatures below 100 °C in what appeared to be a one-step process. Manek and Kolling^45^ reported the PXRD of the final dehydration product, which is a good match for the form 1 (F1) anhydrate structure determined several years later (HEBFUR, *P*2_1_/*c*: *a* = 13.485 Å, *b* = 7.067 Å, *c* = 13.510 Å, β = 98.34°).^27^ Our analyses of H_A_ were in good agreement with these previous reports. DSC thermograms (unsealed pans) of as-grown material have two endothermic transitions (Figure S2). The first, with a *T*_max_ = 91.4 ± 0.2 °C, can be attributed to dehydration and the higher one at 230.3 ± 0.1 °C, to melting of the anhydrate. Ground samples heated under the same conditions had a comparable dehydration *T*_max_ = 88.7 ± 2.7 °C. TGA weight loss for H_A_ was 5.1 ± 0.1% (calcd. = 5.2%), with rapid water loss between approximately 55 and 85 °C when heated at a standard rate of 5 °C/min (Figure 4B).

The mechanism and kinetics of the solid state transformation from H_A_ to F1 anhydrate were investigated using complementary sPXRD and isothermal TGA methods (both RH% = 0). All time-resolved *in situ* sPXRD experiments were performed on phase-pure H_A_ using a controlled atmosphere flow cell and a data acquisition method that enabled high resolution patterns to be collected every ∼20 s. A representative contour plot tracking the structural changes that occur when H_A_ was heated at 6 °C/min from room temperature to ∼190 °C is shown in Figure 4A. For discussion purposes, we will consider the structural changes in three distinct temperature regions: below 155 °C (stage 1), between 155–165 °C (stage 2), and above 165 °C (stage 3).

**Figure 5:**
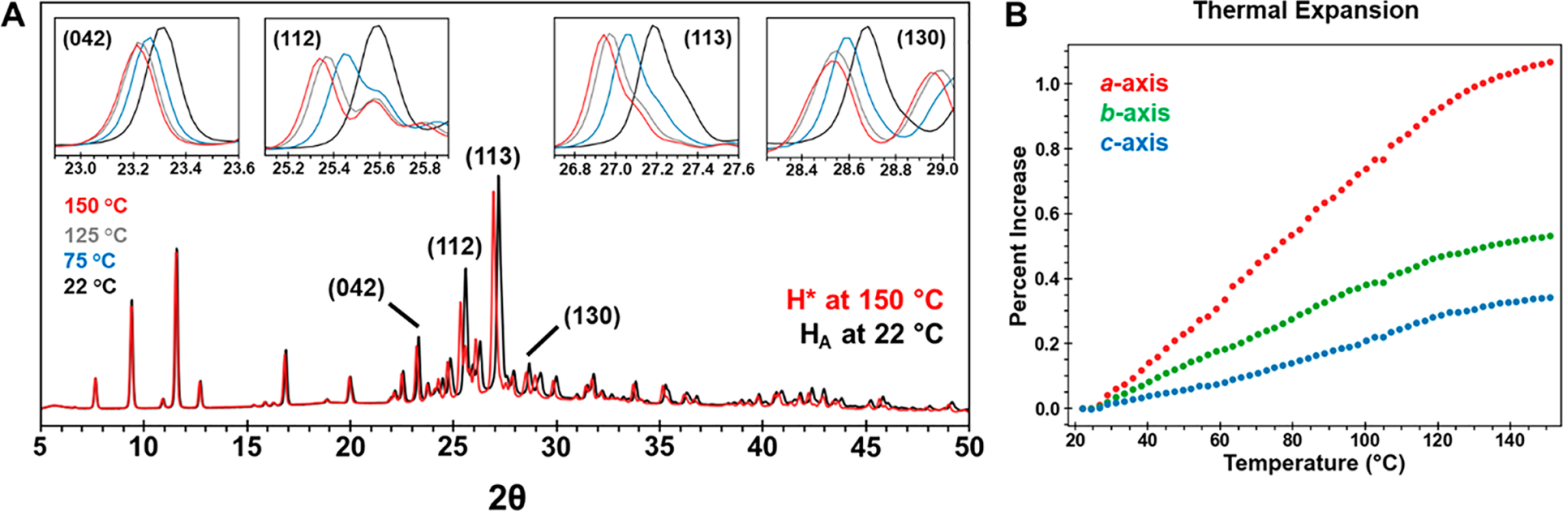
Stage 1 of the H_A_ dehydration process involves water loss and the formation of isomorphous desolvate H*. (A) Comparison of the sPXRD patterns at 22 and 150 °C shows the change in intensity for select (*hkl*) reflections. Inset images are close up views of (042), (112), (113), and (130) diffraction lines at 22, 75, 125, and 150 °C. All show a decrease in intensity due to water loss and a shift to slightly lower 2θ values due to thermal expansion. (B) Change in lattice cell axes determined from Pawley refinement of sPXRD data.

In stage 1, both the absence of major changes in the PXRD pattern between 22 and 155 °C and the subtle changes in this temperature region are instructive. TGA indicated that dehydration of H_A_ is essentially complete by 100 °C, yet the H_A_ lattice persists to at least 150 °C. Dehydration of H_A_ must therefore initially yield an isomorphous desolvate (H*). Water loss from the H_A_ lattice is also evidenced by changes in the relative intensities of select PXRD diffraction lines over time and as the temperature increases (Figures 5a, S3). Comparison of the simulated PXRD patterns of H_A_ (OBEQAN01) and a modified CIF file with the water molecules removed predicted a significant decrease in the intensity of (112) due to the change in electron density in that plane. Experimental data confirmed that the intensity of this peak decreased by 18.5% between 22 and 75 °C. Additional diffraction lines such as (113), (042), and (130) also showed decreases of 6.4%, 6.1%, and 5.0%, respectively. Temperature changes can also contribute to slight changes in peak intensities, though it is difficult to distinguish between the two effects.

Peak positions also shift slightly due to thermal expansion effects. Sequential Pawley refinement of sPXRD patterns collected between 22 and 150 °C showed no abrupt changes indicative of a phase change (Figures 5b, S4). From this, we conclude that water loss occurs gradually over a broad temperature range. The thermal expansion was notably anisotropic with a much larger increase along the *a* axis (0.7%), the π-stacking direction, relative to the *b* axis (0.34%) or *c* axis (0.22%). The cell volume increase over this temperature range was ∼2.0%.

**Figure 6:**
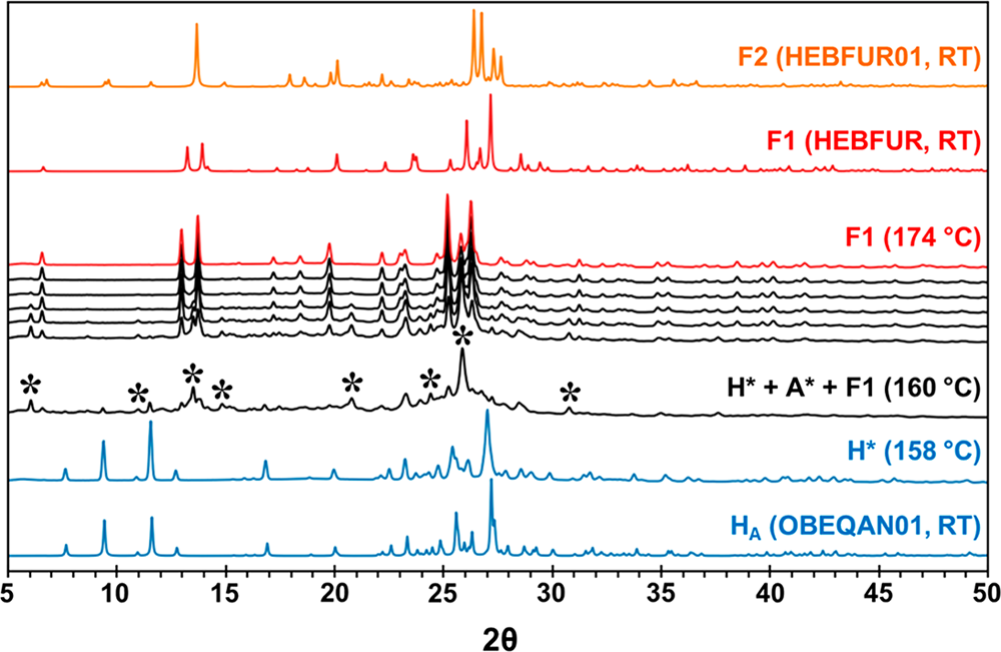
Stage 2 sPXRD patterns between 158 and 174 °C. All diffraction lines at 158 °C correspond to H*. The sPXRD pattern at 160 °C has diffraction lines corresponding to H*, F1, and at least eight other diffraction lines (asterisked) associated with A*, which is likely a mixture of two or more phases. By 174 °C, all diffraction lines correspond to F1.

With continued heating, H* undergoes a dramatic structural change in stage 2. In the experiment shown in Figure 4, all diffraction lines in the 158 °C sPXRD pattern can be attributed to H*. Yet by 160 °C (20 s later), only small amounts of H* remained, and several new diffraction lines appeared. Several of the new low-intensity peaks have 2θ values that match anhydrate F1, though the most intense peaks do not. At least eight diffraction lines in the 160 °C pattern do not correspond to H* or F1 (Figure 6). The unassignable peaks at 2θ = 6.045°, 10.988°, 13.518°, 14.837°, 20.784°, 24.418°, 25.862°, and 30.776° are indicated with an asterisk. We refer to this phase as A*. Attempts to fit these peaks to a unit cell were not successful, possibly because A* is a mixture of two or more phases. The two most intense A* diffraction lines have 2θ values close to those expected for the most intense peaks of the form 2 (F2) anhydrate, but there are insufficient data to make a confident phase assignment.

Some variability in the onset temperature for the transformation of H* was observed in experiments performed under similar conditions, but H* was always stable to at least ∼140 °C. In one experiment, H_A_ was heated to 150 °C, and the H* lattice remained stable at that isothermal temperature for at least 30 min. In another experiment, H* began to convert to F1 below 150 °C but without any apparent A* (Figure S5). This suggests that H* can also convert directly to F1. It is difficult to pinpoint exactly what instability factors trigger the transformation of H* in a given experiment; however, once F1 appears it quickly becomes the dominant phase.

In stage 3, all peaks correspond to F1. For the experiment shown in Figure 4, all of the A* peaks have disappeared by 174 °C. Continued heating of phase pure F1 to a maximum temperature of 192 °C did not reveal any further changes in the PXRD pattern.

### H_A_ Dehydration Kinetics and Mechanism

**Figure 7:**
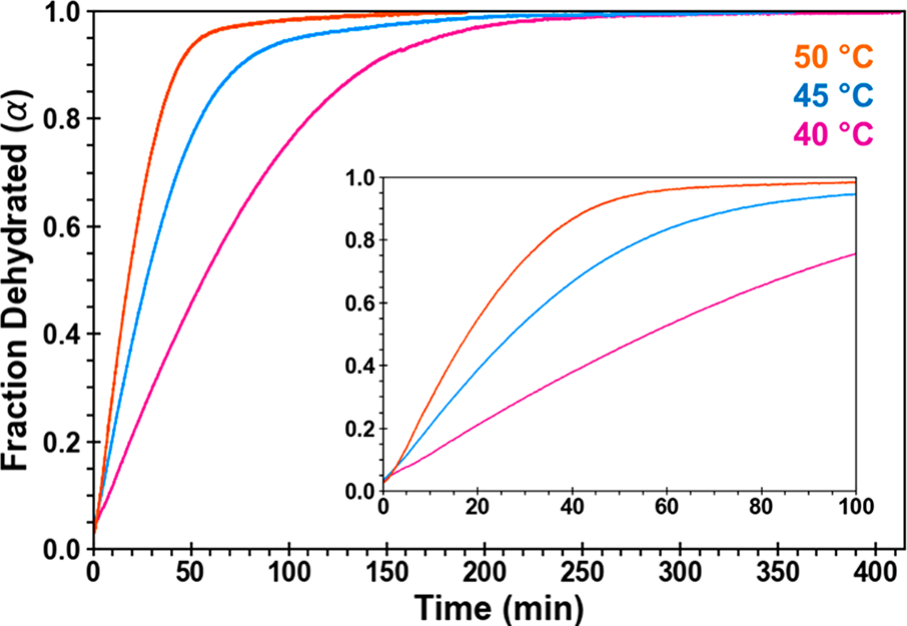
Fraction dehydrated (α) vs time plots for H_A_ based on triplicate isothermal TGA data collected at 40, 45, and 50 °C. Inset plot is an expanded view of the data from 0 to 100 min. All samples were ground and heated at 5 °C/min.

As a complement to sPXRD studies, isothermal TGA kinetics experiments on H_A_ were carried out at 40, 45, and 50 °C. Experiments were run in triplicate, and a representative plot of the fraction dehydrated (α) vs time is shown in Figure 7. The reaction conversion data in the linear region 0.1 < α < 0.9 at each isothermal temperature were fit to each of the 17 different solid state reaction models in Table 1. Fitting the kinetic data often yields one model with a much higher correlation coefficient (*R*^2^) than the others, and this can be informative as to the rate limiting step in the reaction. However, the H_A_ data fit equally well (*R*^2^ values > 0.99) to four very different models: A2 (1D nucleation), R2 and R3 (2D and 3D geometrical contraction), and D1 (1D diffusion; Table S1). While TGA data on their own would be inconclusive, the sPXRD established that dehydration of H_A_ creates isomorphic desolvate H*. Since the measured TGA weight loss corresponds to the H to H* transition, 1D diffusion is the only feasible mechanism of the four.

At 40, 45, and 50 °C, the rate constants for the dehydration reaction were 0.004, 0.008, and 0.012 min^–1^, respectively. These measurements fit to an Arrhenius plot yielded an *E*_a_ = 92.3 ± 24 kJ/mol. The *E*_a_ at different time points throughout the reaction were also calculated using model-free Friedman and standard analysis methods. Both methods indicated a consistent activation energy throughout the dehydration process, which would be expected for a diffusion process (Figure S6). Notably, the measured *E*_a_ corresponds only to the H to H* transformation since the polymorph conversion from H* to F1 has no associated weight loss. Even in the DSC, there was no observable thermal transition above 100 °C prior to melting to suggest an intermediate phase. It is only from the combination of sPXRD and TGA that H_A_ dehydration can be recognized as a two-step process.

### The H* to F1 Polymorph Transformation

**Figure 8:**
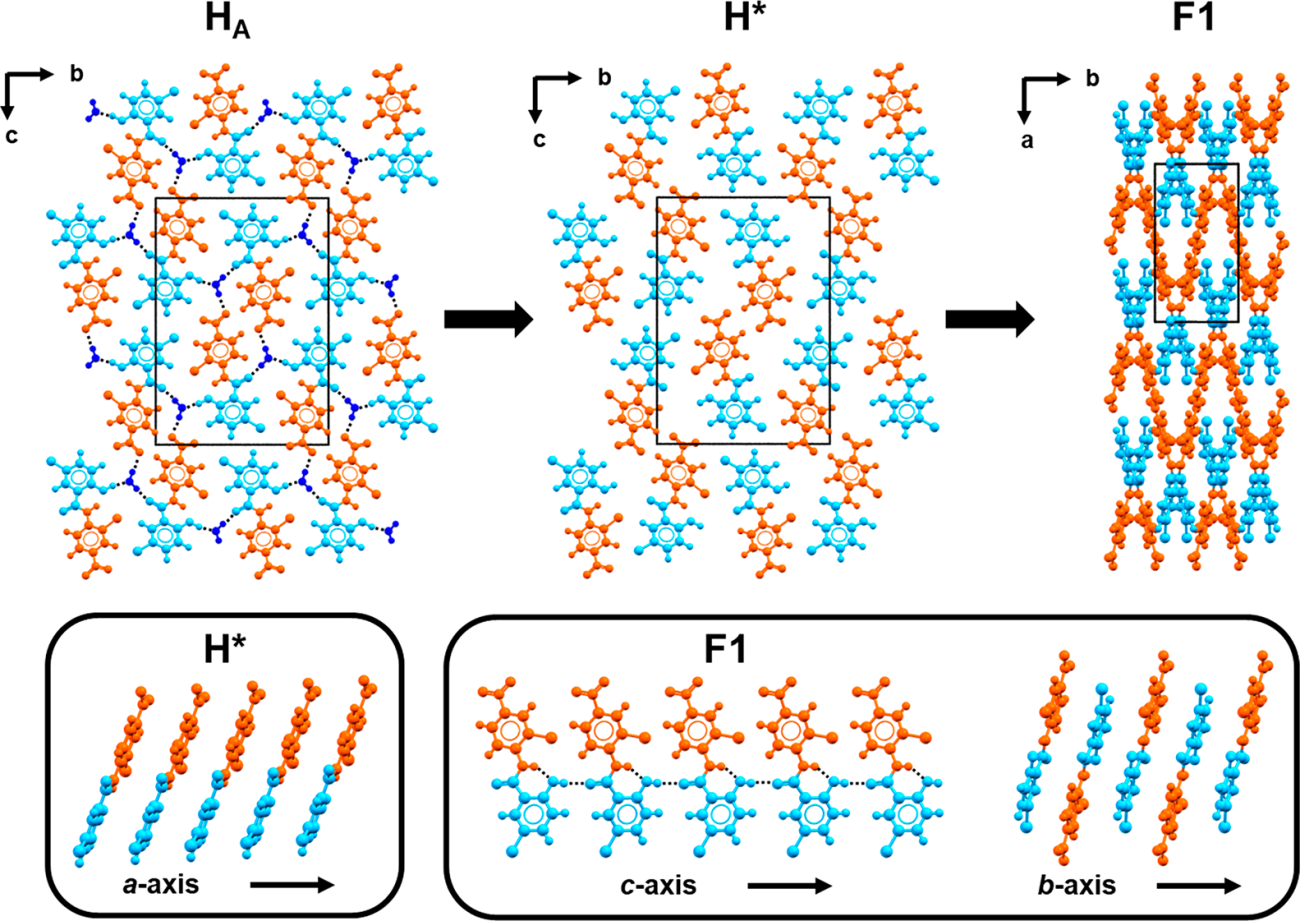
Schematic of the dehydration pathway of H_A_ to F1. Each NCL molecule is colored so that the nitrophenyl ring is orange and the phenol ring is light blue. Water molecules in H_A_ are dark blue.

With knowledge that water loss precedes lattice rearrangement, it becomes possible to propose a molecular-level model to rationalize the H* to F1 transformation pathway that considers the topology in both the π-stacking and hydrogen bonding motifs in the two anhydrous forms. In Figure 8, NCL nitrophenyl rings are colored orange, and the phenol rings are light blue. In H_A_ (and H*), NCL molecules exist in homogeneous face-to-face π-stacks along the *a* axis. The *a* axis is also the direction of highest thermal expansion, such that upon heating, the repeat distance within the stack increases from 3.81 Å (at 22 °C) to 4.07 Å (at 145 °C). Water loss from H_A_ also ruptures the hydrogen bonding interactions, leaving NCL donor (hydroxyl) and acceptor (carbonyl) groups unsatisfied.

In F1, molecules assemble into one-dimensional hydrogen bonded chains (graph set C(6)) of hydroxyl and carbonyl groups along the *c* axis. Molecules along the chain twist by 30.5° presumably to avoid steric interactions, though nitrophenyl and phenol rings are on opposite sides of the hydrogen bonded chain. Assuming that the least motion arguments apply here, as the distance between π-stacked rings increases in H*, it seems likely that eventually it becomes possible for molecules to rotate and hydrogen bond to the neighboring molecule in the stack, thereby initiating the cooperative formation of the C(6) chain. Such a model would effectively mean the *a* axis of H* becomes the *c* axis of F1. This type of cooperative motion is also consistent with the formation of heterogeneous π-stacks in F1 where molecules are related by a 2-fold rotation along the *b* axis. The proposed transformation pathway requires a considerable amount of molecular motion and a volume change. However, all other alternatives such as formation of the C(6) chain from molecules in adjacent stacks would require a significantly greater net reorganization since neighboring stacks in H* are related by a glide plane or 2-fold rotation.

### Dehydration of H_B_

The structure of H_B_ has been known since 1998; however, literature reports on its thermal stability vary greatly. Van Tonder et al.^46^ reported that TGA weight loss due to dehydration occurs in two distinct stages at 173 ± 5 °C and 201 ± 5 °C, while the thermogram presented in Manek and Kolling^45^ shows more gradual weight loss in a lower temperature range between approximately 100 and 175 °C. In our own characterization of H_B_, we observed significant differences in the thermal properties depending on the sample preparation.

**Figure 9:**
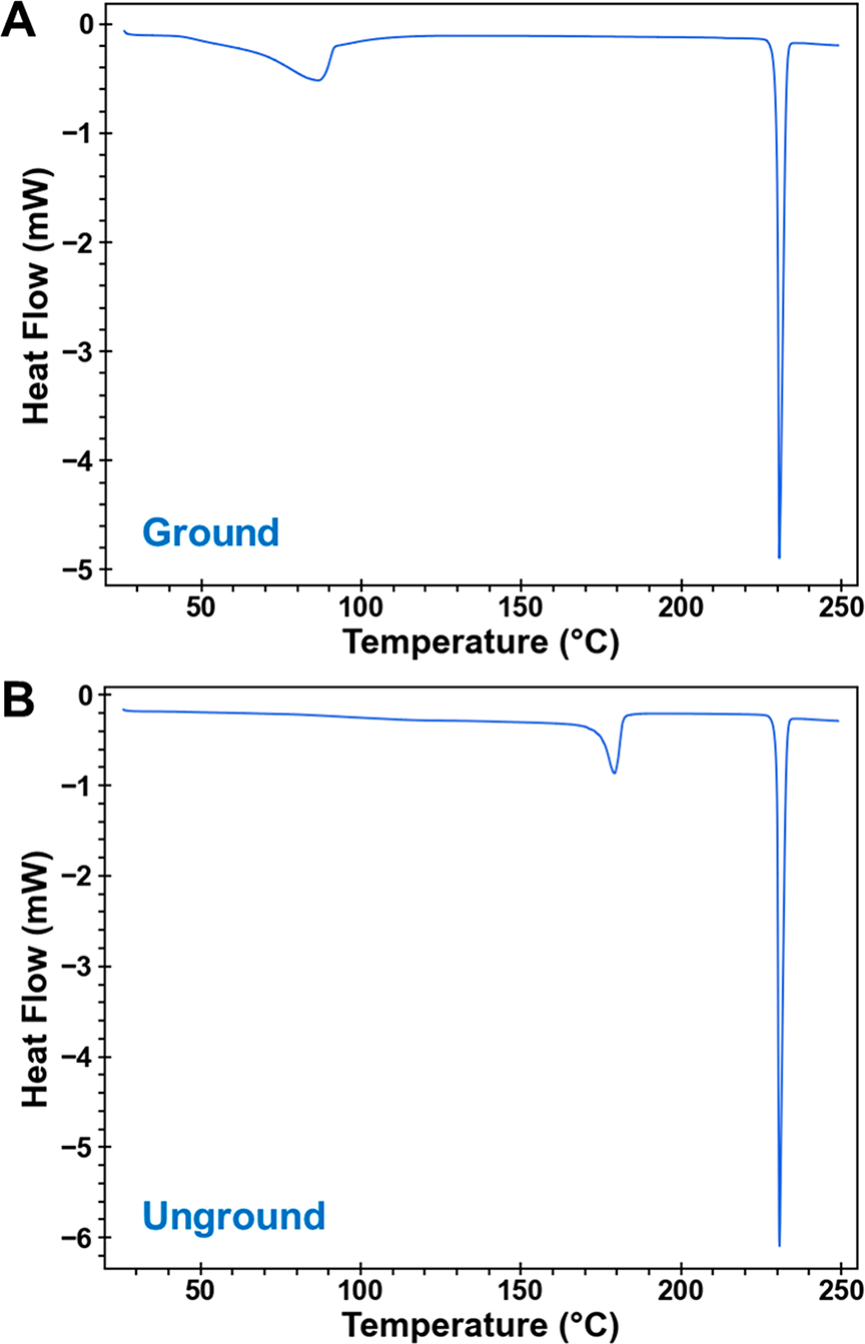
DSC curves of heat flow vs temperature for H_B_ samples (A) ground with a mortar and pestle and (B) unground. Samples were heated at 5 °C/min to 250 °C with unsealed lids.

DSC analyses of as-grown (unground) H_B_ had two endotherms, the first with a *T*_max_ = 178.5 ± 0.7 °C corresponding to dehydration and the second at 231 ± 0.2 °C to melting of the anhydrate (Figure 9). If the same H_B_ sample was ground, the first endothermic transition occurred at a dramatically lower temperature, *T*_max_ = 88.7 ± 2.7 °C. TGA analysis of ground H_B_ showed that weight loss begins around 50 °C, declines steeply between 55 and 80 °C, and is complete around 100 °C (Figure S7). Total weight loss for ground H_B_ was 4.8% ± 0.1% (theoretical = 5.2%). The DSC and TGA results clearly show that grinding induces a dramatic decrease in the thermal stability of H_B_.

**Figure 10:**
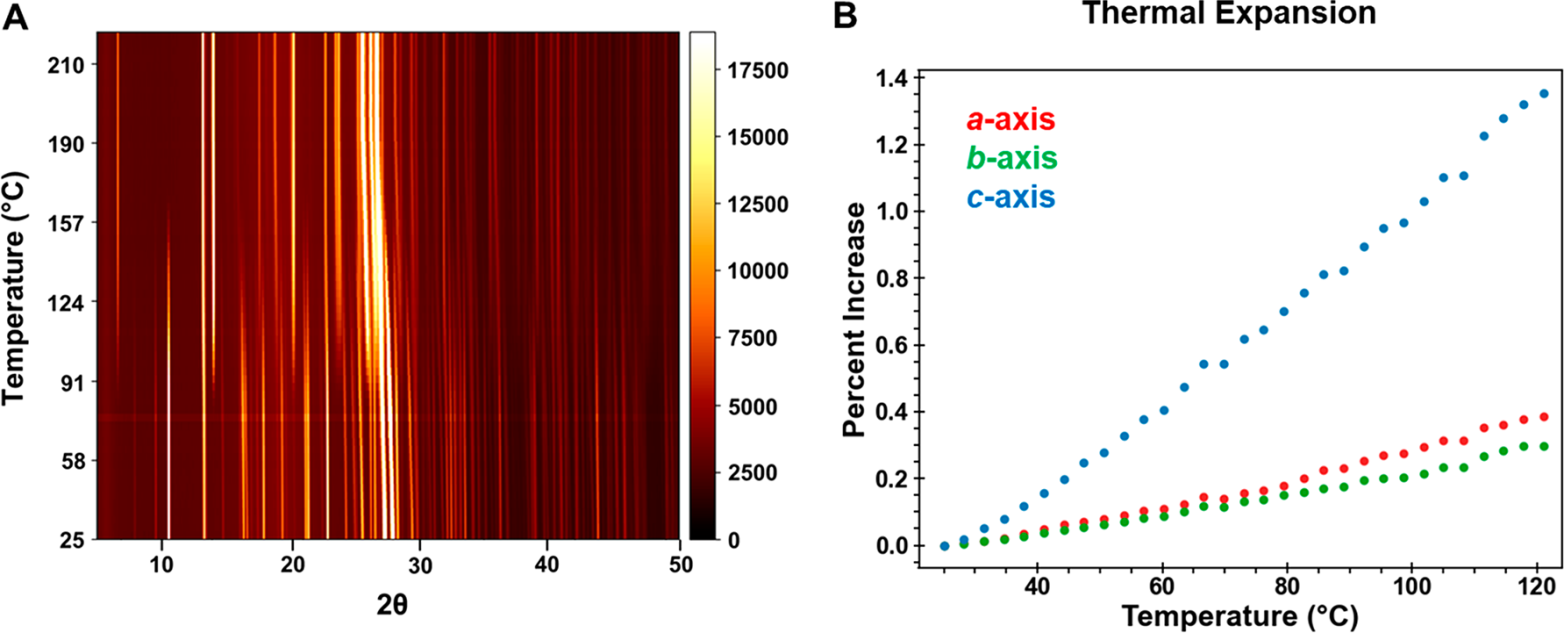
(A) Time-resolved sPXRD of ground H_B_ heated at 10 °C/min from 25 to 210 °C. The 2θ scale is normalized to Cu Kα. F1 first appears at 76 °C and gradually increases in intensity over the next 10 min until the transformation is complete. (B) Thermal expansion of cell axes in H_B_ determined from sequential Pawley refinement of sPXRD patterns.

A time-resolved *in situ* synchrotron PXRD was used to track the structural changes that occur when H_B_ was dehydrated at a heating rate of 10 °C/min. A representative contour plot is shown in Figure 10. Peaks corresponding to F1 are first observed at approximately 76 °C. Over the next ∼10 min, the F1 peaks gradually increase in intensity while the H_B_ peaks correspondingly decrease and completely disappear by 174 °C. Notably, there was no evidence of any other intermediate crystalline phases in the transformation process. H_B_ thermal expansion in the 25–120 °C range was highly anisotropic, with a significantly greater increase along the π-stacking *c*-axis direction (∼1.35%) compared to the *a* and *b* axes, which both expanded by <0.4%. The cell volume increase over this temperature range was ∼2.15%.

In isothermal sPXRD experiments, when H_B_ was held at temperatures from 30 to 40 °C, the transformation to F1 also occurred gradually, albeit over much longer time periods. At 35 °C, small amounts of H_B_ persisted in the sample even after 8 h. At 40 °C, conversion to F1 was complete in about 3 h. For more accurate kinetics, isothermal TGA experiments were performed on ground H_B_ at 40, 45, and 50 °C (Figure S9). The fraction dehydrated (α) vs time was determined based on weight change, and the data from the linear region 0.1 < α < 0.8 were fit to each of the 17 different solid state reaction models (Table S2). With an *R*^2^ > 0.999, the geometrical contraction models R3 and R2 were the top fits at 40 °C. R3 and F1 were the best fits at 45 and 50 °C (*R*^2^ > 0.996). Geometrical contraction models mathematically describe a reaction mechanism where the nucleation of the product on the surface(s) of the reacting particles is the rate limiting step. R2 and R3 models simply differ in how they approximate particle shape, with the former assuming a cylinder and the latter a sphere.

When the kinetic data were fit to the R3 model, the calculated *E*_a_ = 77.9 ± 8.2 kJ/mol. *E*_a_ values calculated with R2 and F1 models were similar. Both Friedman and standard model-free analysis methods indicated that the *E*_a_ decreases over the course of the reaction (Figure S10). This is consistent with a surface-limited reaction model, because the area of the reacting front progressively decreases as H_B_ transforms.

With knowledge that the transformation of H_B_ to F1 proceeds via a surface-mediated mechanism, we return to the question of why ground and unground materials have such vastly different DSC dehydration temperatures (Figure 9). Several possible explanations were considered. We initially hypothesized that grinding may create small amounts of F1, which might seed the transformation. However, careful analysis of the sPXRD patterns from three different ground batches of H_B_ revealed no crystalline phase impurities in the samples (Figure S11). Similarly, while grinding of any organic material may yield small amounts of amorphous material, there was also no appreciable amorphous content in the sPXRD patterns. While it is possible that grinding creates uniquely reactive defect sites, it is less clear how such sites can be identified or characterized.

Having excluded phase impurities, we considered the surfaces that are created during particle breakage. Particle size effects^51^ are often used to explain a decrease in the dehydration temperature of a few degrees; however, manual grinding lowered the *T*_max_ of H_B_ by nearly 90 °C. We are not aware of any literature precedent for a decrease of this magnitude. Hot stage microscopy of unground H_B_ showed that all crystal surfaces appear to darken fairly uniformly, such that there was no obvious anisotropy in the dehydration (Figure S12). Though it was not possible to reliably assign Miller indices to the faces of ground crystals, slip planes calculated using the CSD-Particle module^52^ essentially all bisect the *c* axis (needle axis; Figure S13). Assuming particle fracture is most likely to occur along these low rugosity planes, increased mobility of NCL molecules on these higher index surfaces may play a critical role in lowering the nucleation barrier of F1.^53^ Additional experiments beyond the scope of this study would be needed to confirm this hypothesis.

## Conclusions

H_A_ and H_B_ both dehydrate to the same final dehydration product, F1, but through a combination of time-resolved synchrotron diffraction and thermogravimetric analysis, their dehydration pathways were found to be distinctly different. Diffusional water loss from H_A_ yields a lower density isomorphous desolvate, H*, an intermediate which exhibits remarkable stability to temperatures at least 40−50 °C above water’s boiling point. A H* to F1 model was proposed to account for the transformation pathway wherein molecules within a π-stack direction rotate to form one-dimensional hydrogen bonded chains along an axis orthogonal to the original π-stacking direction. The least-motion transformation model is supported by thermal expansion data which indicated the largest increase along the π-stacking direction.

Unlike H_A_, the lattice topologies and symmetry within the π-stacks in H_B_ and F1 are quite different, such that there is no obvious least-motion pathway to convert between the two (Figure S14). The absence of crystalline intermediates in the time-resolved sPXRD dehydration experiments and the thermogravimetric kinetic analyses point to a mechanism wherein surface recrystallization is the rate-limiting step in the transformation of H_B_ to F1. The decrease in the thermal stability of H_B_ upon manual grinding is quite remarkable. While a definitive molecular-level explanation for this phenomenon remains elusive, it seems probable that grinding creates low-rugosity faces which help to facilitate the surface-mediated transformation of H_B_ to F1.

## Supporting Information Available

Micrographs, TGA, DSC, synchrotron PXRD, kinetics tables (PDF)
