# Supplementary material for: Dehydration of Niclosamide Monohydrate Polymorphs: Different Mechanistic Pathways to the Same Product
Source: Cryst Growth Des. 2023 Jun 2;23(7):5102–11. doi: 10.1021/acs.cgd.3c00322 (PMC10950297; doi:10.1021/acs.cgd.3c00322)
Supplement: Supplementary file 1 — cg3c00322_si_001.pdf [file cg3c00322_si_001.pdf]

Supporting Information

Dehydration of Niclosamide Monohydrate Polymorphs: Different Mechanistic Pathways to the Same Product

Jen E. Mann, Renee Gao, and Jennifer A. Swift*

Georgetown University, Department of Chemistry, Washington, DC 20057-1227

Table of Contents **Page**

Figure S1. Optical images of (left) H_A and (right) H_B taken at the same magnification. Both monohydrates exhibit a needle-like morphology. H_A crystallizes as fine needles, while H_B crystallizes as clusters of thick needles. Scale bars = 500 μm. 3

Figure S2. DSC curves of heat flow vs. temperature for H_A samples that were (A) ground with a mortar and pestle and (B) unground. DSC samples were heated at 5 °C/min to 250 °C in aluminum pans with unsealed lids. 3

Figure S3. Change in peak intensity for (130), (042), (113), and (112) diffraction lines with temperature. From 22 to 75 °C, the peaks decrease by 5.0%, 6.1%, 6.4% and 18.5%, respectively. 4

Figure S4. Change in cell volume and beta angle of the H_A (and/or H*) lattice from 22 to 150 °C determined from Pawley refinement of sPXRD data. 4

Figure S5. Contour plots of two additional H_A dehydration experiments performed under similar heating conditions. (Left) H_A was heated to 150 °C and held isothermally for 30 minutes. H* remained stable. (Right) The H* to F1 conversion occurred below 150 °C. 5

Table S1. Regression parameters associated with different solid state reaction models for isothermal TGA data (40, 45, and 50 °C) for H_A (ground). Reaction models with the highest statistical fit are indicated, with $R^2 > 0.99$ in red. 6

Figure S6. (A) Summary of E_a determination from three runner-up model-based kinetic analyses of isothermal TGA data on H_A collected at 40, 45 and 50°C. (B) Arrhenius plot. All four models with the highest R^2 coefficients yielded similar E_a values. (C) E_a vs. alpha calculated from the model-free Friedman analysis method. (D) E_a vs. alpha calculated using the standard model-free method. 7

Figure S7. TGA of ground H_B in open pans heated at 5 °C/min. 7

Figure S8. Change in cell volume and beta angle of the H_B lattice from 25 to 120 °C determined from Pawley refinement of sPXRD data. **8**

Figure S9. Fraction dehydrated (α) vs. time plots for H_B based on triplicate isothermal TGA data collected at 40, 45 and 50 °C. Inset plot is an expanded view of the data from 0 – 100 min. All samples were ground and heated at 5 °C/min. **8**

Table S2. Regression parameters associated with different solid state reaction models for isothermal TGA data (40, 45, and 50 °C) for H_B (ground). Reaction models with the highest statistical fit are indicated, with $R^2 > 0.99$ in red. Ones with $R^2 > 0.999$ are in bold. **9**

Figure S10. (A) Summary of E_a determination from two runner-up model-based kinetic analyses of isothermal TGA data on H_B collected at 40, 45 and 50°C. (B) Arrhenius plot. All models with $R^2 > 0.99$ coefficients yielded similar E_a values. (C) E_a vs. alpha calculated from the model-free Friedman analysis method. (D) E_a vs. alpha calculated using the standard model-free method. **10**

Figure S11. sPXRD of three different ground batches of H_B compared against the PXRD patterns of HB and F1 simulated from the single crystal structures. (left) A zoomed view of the region where high intensity peaks would be expected if F1 was present in the samples. **11**

Figure S12. Hot-stage microscopy optical images of H_B ramped at 5 °C/min from room temperature to 150 °C. At approximately 95 °C dark spots begin to appear across the surface until 140 °C whereupon optical changes can no longer be observed. Scale bar = 100 μm. **11**

Figure S13. (left) Calculated BFDH morphology and (right) top ten slip planes calculated using the CSD-Particle module in the Cambridge Structural Database. None of the calculated slip planes correspond to the natural faces. **12**

Figure S14. Schematic of packing in H_B and F1. Each NCL molecule is colored so that the nitrophenyl ring is orange and the phenol ring is light blue. Water molecules in H_B are dark blue. **12**

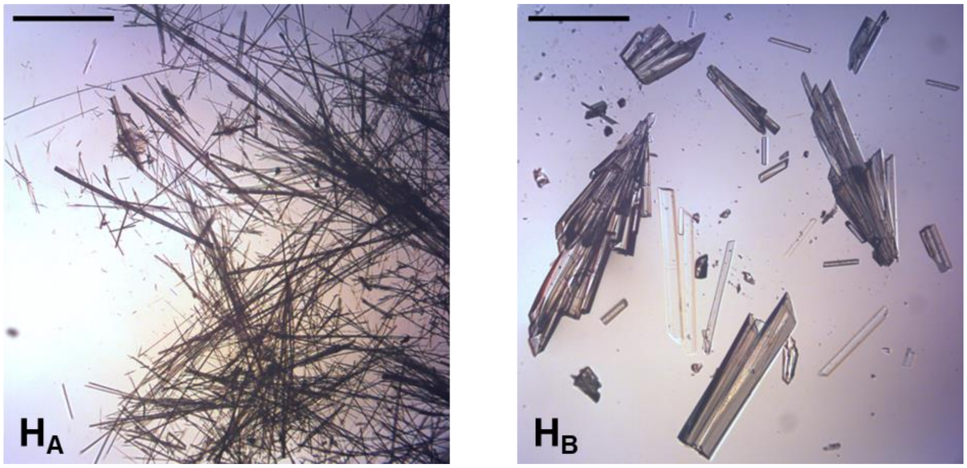

Figure S1. Optical images of (left) H_A and (right) H_B taken at the same magnification. Both monohydrates exhibit a needle-like morphology. H_A crystallizes as fine needles, while H_B crystallizes as clusters of thick needles. Scale bars = 500 μm.

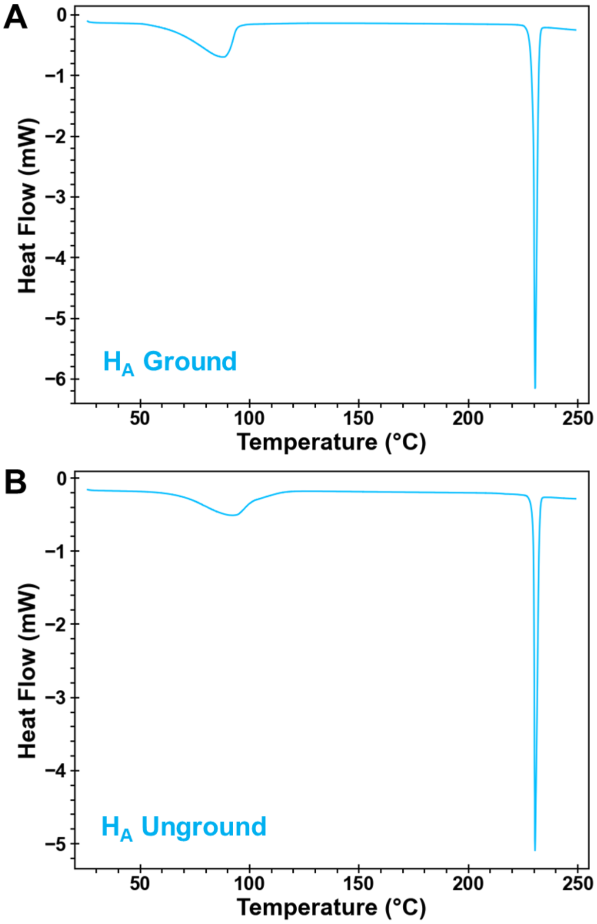

Figure S2. DSC curves of heat flow vs. temperature for H_A samples that were (A) ground with a mortar and pestle and (B) unground. DSC samples were heated at 5 °C/min to 250 °C in aluminum pans with unsealed lids.

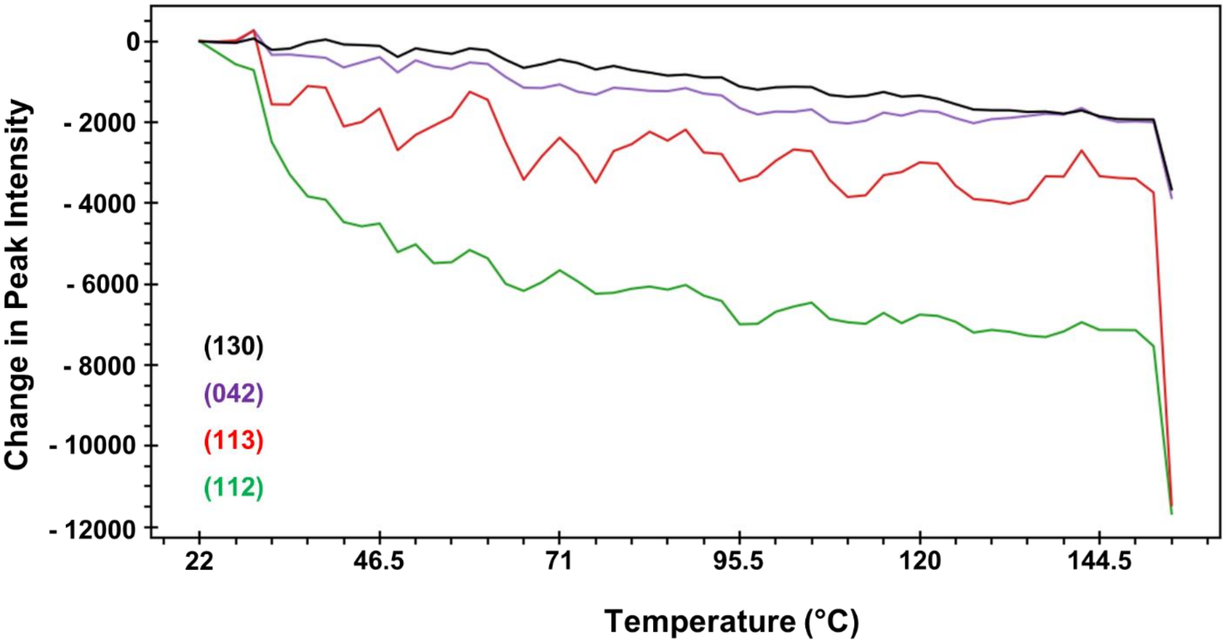

Figure S3. Change in peak intensity for (130), (042), (113), and (112) diffraction lines with temperature. From 22 to 75 °C, the peaks decrease by 5.0%, 6.1%, 6.4% and 18.5%, respectively.

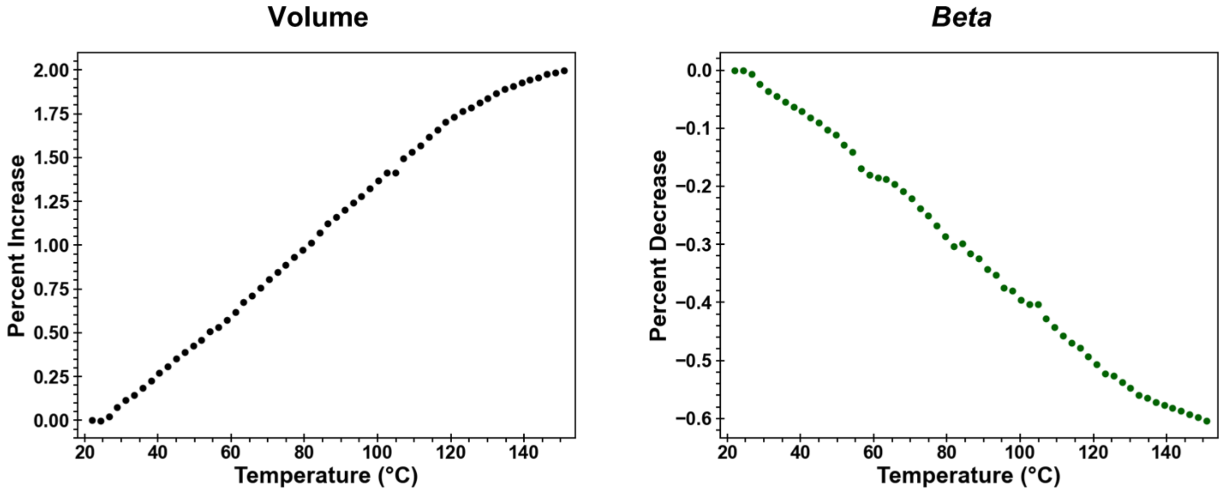

Figure S4. Change in cell volume and beta angle of the H_A (and/or H*) lattice from 22 to 150 °C determined from Pawley refinement of sPXRD data.

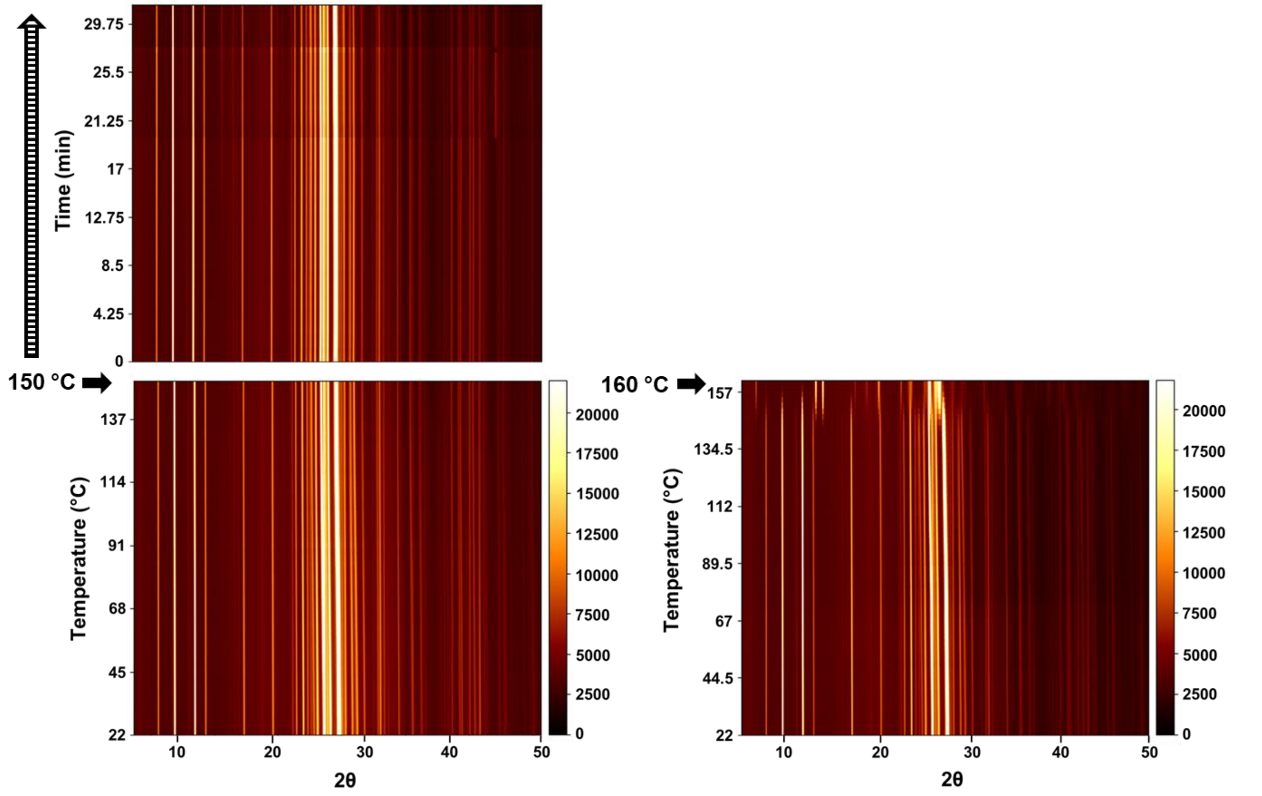

Figure S5. Contour plots of two additional H_A dehydration experiments performed under similar heating conditions. (Left) H_A was heated to 150 °C and held isothermally for 30 minutes. H* remained stable. (Right) The H* to F1 conversion occurred below 150 °C.

Table S1. Regression parameters associated with different solid state reaction models for isothermal TGA data (40, 45, and 50 °C) for H_A (ground). Reaction models with the highest statistical fit are indicated, with $R^2 > 0.99$ in red.

	**40 °C**	**45 °C**	**50 °C**
**Nucleation Models**			
1D growth of nuclei (Avrami-Erofeyev Eq, n = 2) (**A2**)	0.99525	0.99487	0.99584
2D growth of nuclei (Avrami-Erofeyev Eq, n = 3) (**A3**)	0.98554	0.98414	0.98482
3D growth of nuclei (Avrami-Erofeyev Eq, n = 4) (**A4**)	0.97760	0.97573	0.97624
Random nucleation (Prout-Tompkins Eq) (**B1**)	0.98836	0.98719	0.98794
Power law (n = 1/2) (**P2**)	0.93286	0.92775	0.92840
Power law (n = 1/3) (**P3**)	0.91164	0.90591	0.90649
Power law (n = 1/4) (**P4**)	0.89966	0.89360	0.89415
**Geometrical Contraction Models**			
2D phase boundary (Contracting area) (**R2**)	0.99701	0.99687	0.99800
3D phase boundary (Contracting volume) (**R3**)	0.99688	0.99788	0.99906
**Diffusion Models**			
1D diffusion (**D1**)	0.99194	0.99307	0.99419
2D diffusion (**D2**)	0.97794	0.98209	0.98305
3D diffusion (Jander Eq) (**D3**)	0.93565	0.94428	0.94470
3D Diffusion (Ginstling-Brounshtein Eq) (**D4**)	0.96644	0.97206	0.97287
**Reaction Order Models**			
Zero-order (**R1**)	0.97565	0.97243	0.97339
First-order (**F1**)	0.98393	0.98746	0.98865
Second-order (**F2**)	0.85251	0.86649	0.86627
Third-order (**F3**)	0.66572	0.69136	0.68855

H_A dehydration to F_I	Ea (kJ/mol)
**R3** Dehydration Model	92.4 ± 24
**R2** Dehydration Model	92.2 ± 24
**A2** Dehydration Model	92.2 ± 24

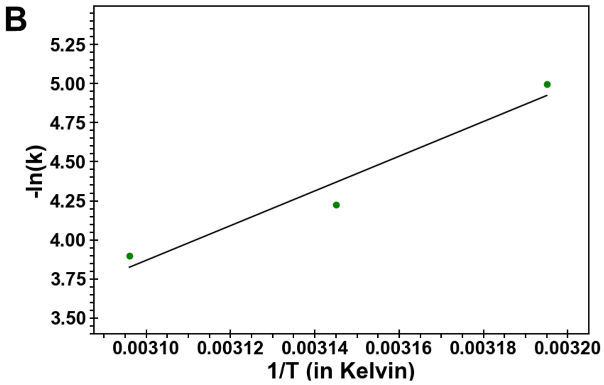

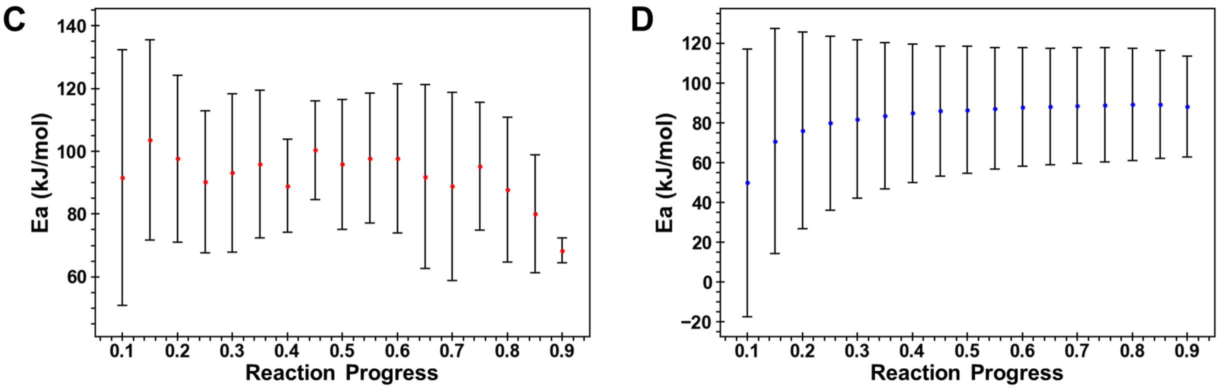

Figure S6. (A) Summary of E_a determination from three runner-up model-based kinetic analyses of isothermal TGA data on H_A collected at 40, 45 and 50°C. (B) Arrhenius plot. All four models with the highest R^2 coefficients yielded similar E_a values. (C) E_a vs. alpha calculated from the model-free Friedman analysis method. (D) E_a vs. alpha calculated using the standard model-free method.

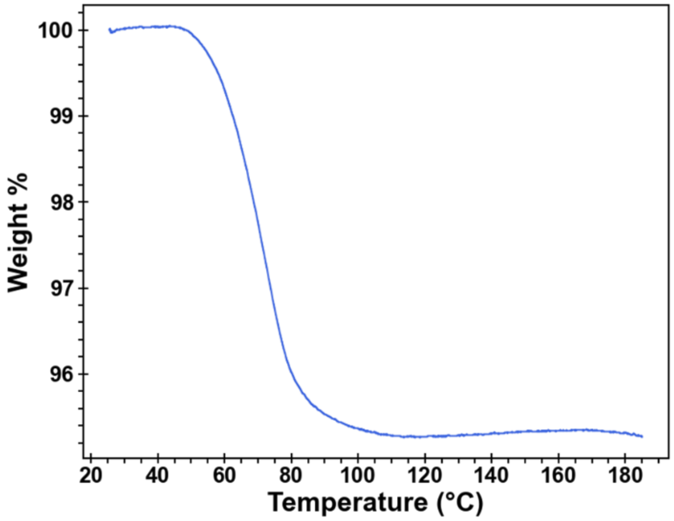

Figure S7. TGA of ground H_B in open pans heated at 5 °C/min.

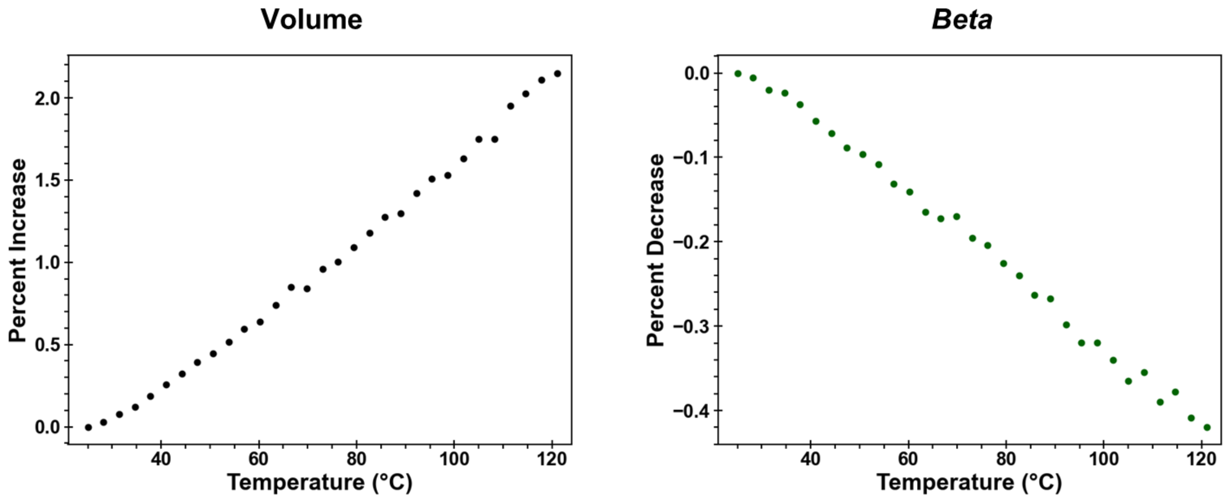

Figure S8. Change in cell volume and beta angle of the H_B lattice from 25 to 120 °C determined from Pawley refinement of sPXRD data.

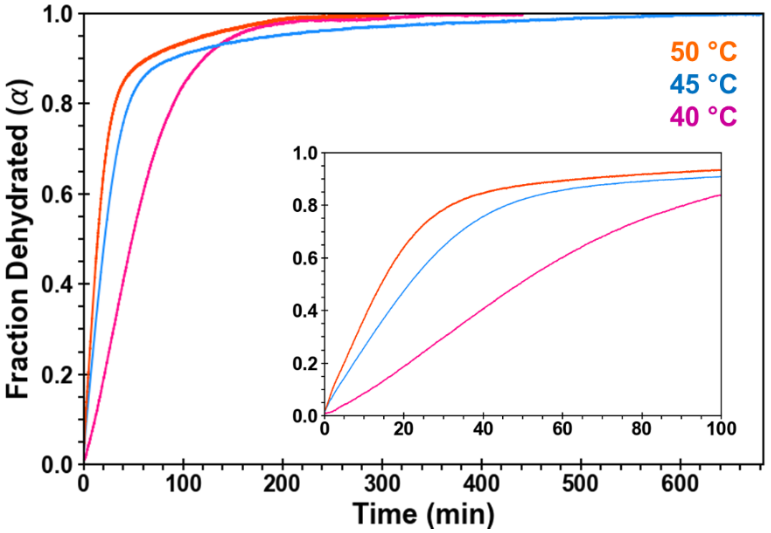

Figure S9. Fraction dehydrated (α) vs. time plots for H_B based on triplicate isothermal TGA data collected at 40, 45 and 50 °C. Inset plot is an expanded view of the data from 0 – 100 min. All samples were ground and heated at 5 °C/min.

Table S2. Regression parameters associated with different solid state reaction models for isothermal TGA data (40, 45, and 50 °C) for H_B (ground). Reaction models with the highest statistical fit are indicated, with $R^2 > 0.99$ in red. Ones with $R^2 > 0.999$ are in bold.

	**40 °C**	**45 °C**	**50 °C**
**Nucleation Models**			
1D growth of nuclei (Avrami-Erofeyev Eq, n = 2) (**A2**)	0.99551	0.98342	0.98556
2D growth of nuclei (Avrami-Erofeyev Eq, n = 3) (**A3**)	0.98544	0.96816	0.97131
3D growth of nuclei (Avrami-Erofeyev Eq, n = 4) (**A4**)	0.97800	0.95825	0.96199
Random nucleation (Prout-Tompkins Eq) (**B1**)	0.98756	0.97178	0.97473
Power law (n = 1/2) (**P2**)	0.95525	0.92620	0.93163
Power law (n = 1/3) (**P3**)	0.93763	0.90561	0.91217
Power law (n = 1/4) (**P4**)	0.92753	0.89409	0.90125
**Geometrical Contraction Models**			
2D phase boundary (Contracting area) (**R2**)	**0.99919**	0.99180	0.99289
3D phase boundary (Contracting volume) (**R3**)	**0.99909**	0.99573	0.99642
**Diffusion Models**			
1D diffusion (**D1**)	0.98996	0.99368	0.99312
2D diffusion (**D2**)	0.97509	0.98915	0.98804
3D diffusion (Jander Eq) (**D3**)	0.94598	0.97205	0.97073
3D Diffusion (Ginstling-Brounshtein Eq) (**D4**)	0.96649	0.98471	0.98349
**Reaction Order Models**			
Zero-order (**R1**)	0.98851	0.96955	0.97224
First-order (**F1**)	0.99300	0.99786	0.99794
Second-order (**F2**)	0.92988	0.95964	0.95921
Third-order (**F3**)	0.82239	0.87390	0.87413

A

H_B dehydration to F_I	Ea (kJ/mol)
**F1** Dehydration Model	79.0 ± 9.0
**R2** Dehydration Model	77.4 ± 7.8

B

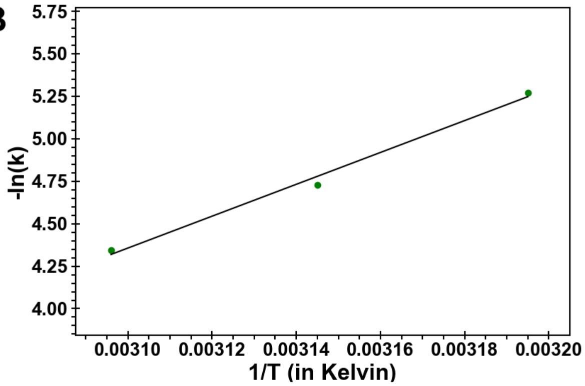

C

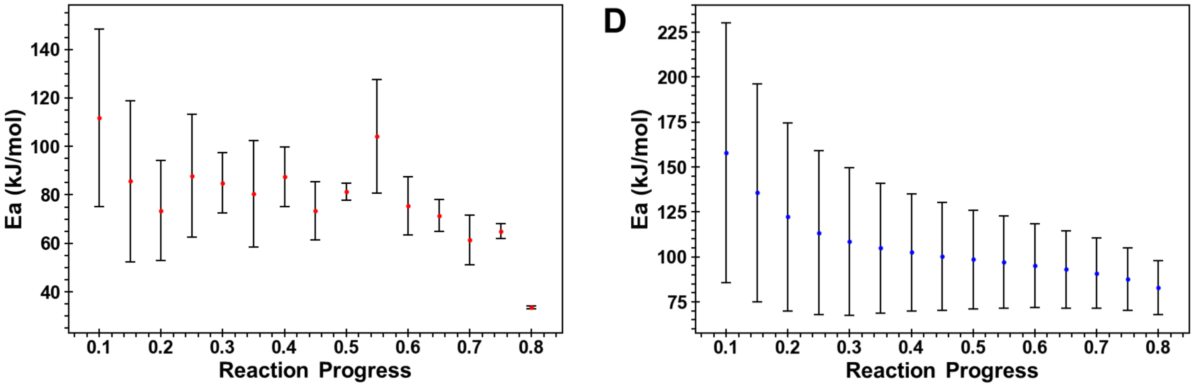

Figure S10. (A) Summary of E_a determination from two runner-up model-based kinetic analyses of isothermal TGA data on H_B collected at 40, 45 and 50°C. (B) Arrhenius plot. All models with $R^2 > 0.99$ coefficients yielded similar E_a values. (C) E_a vs. alpha calculated from the model-free Friedman analysis method. (D) E_a vs. alpha calculated using the standard model-free method.

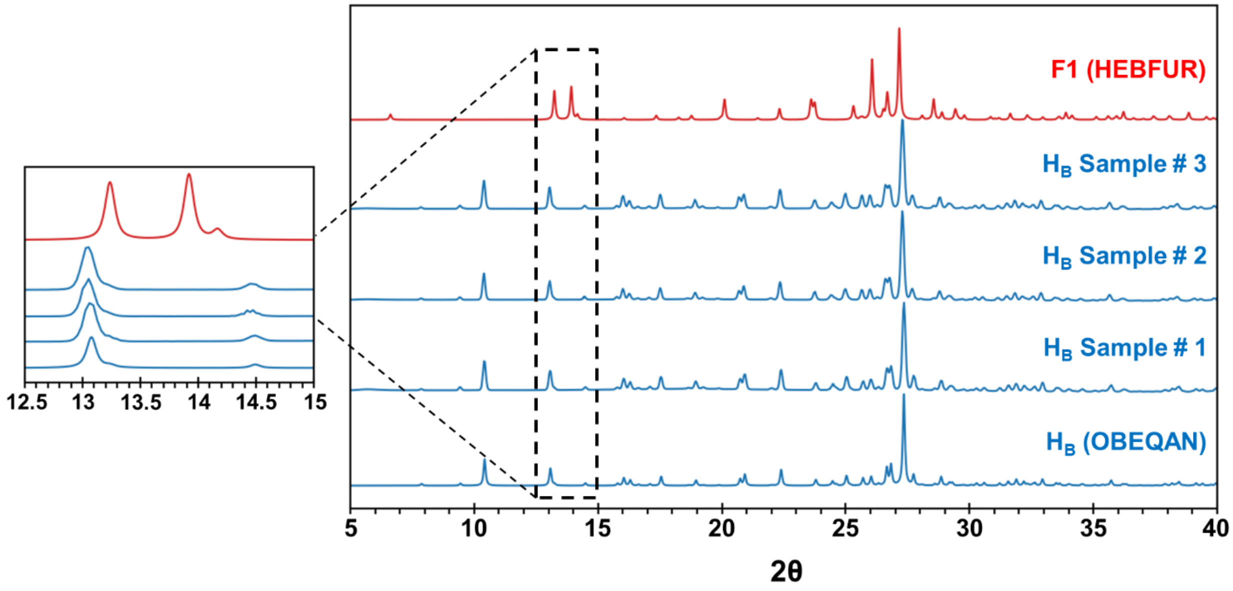

Figure S11. sPXRD of three different ground batches of H_B compared against the PXRD patterns of HB and F1 simulated from the single crystal structures. (left) A zoomed view of the region where high intensity peaks would be expected if F1 was present in the samples.

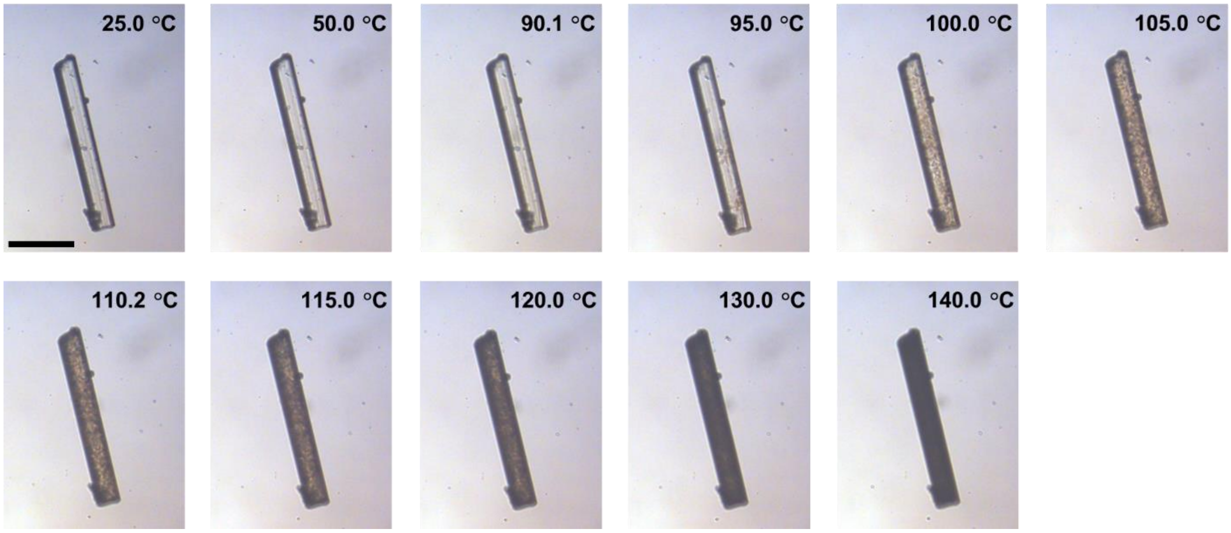

Figure S12. Hot-stage microscopy optical images of H_B ramped at 5 °C/min from room temperature to 150 °C. At approximately 95 °C dark spots begin to appear across the surface until 140 °C whereupon optical changes can no longer be observed. Scale bar = 100 µm.

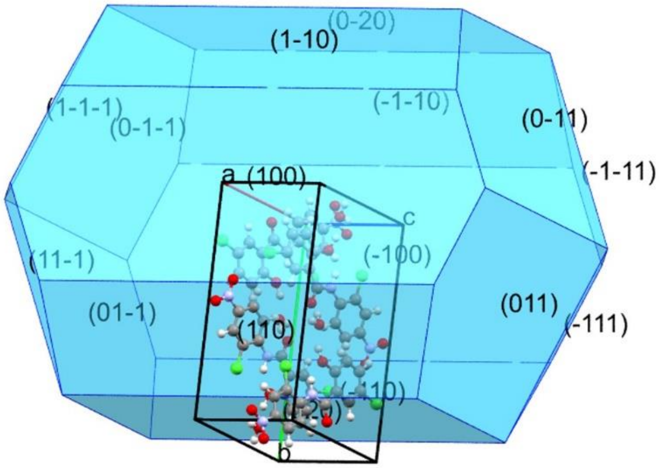

Top ten slip planes calculated with CSD-Particle

(1,0,2)	(3,0,8)
(1,0,4)	(3,1,9)
(2,1,5)	(3,-1,9)
(2,-1,5)	(2,0,6)
(1,-1,4)	(3,0,8)

Figure S13. (left) Calculated BFDH morphology and (right) top ten slip planes calculated using the CSD-Particle module in the Cambridge Structural Database. None of the calculated slip planes correspond to the natural faces.

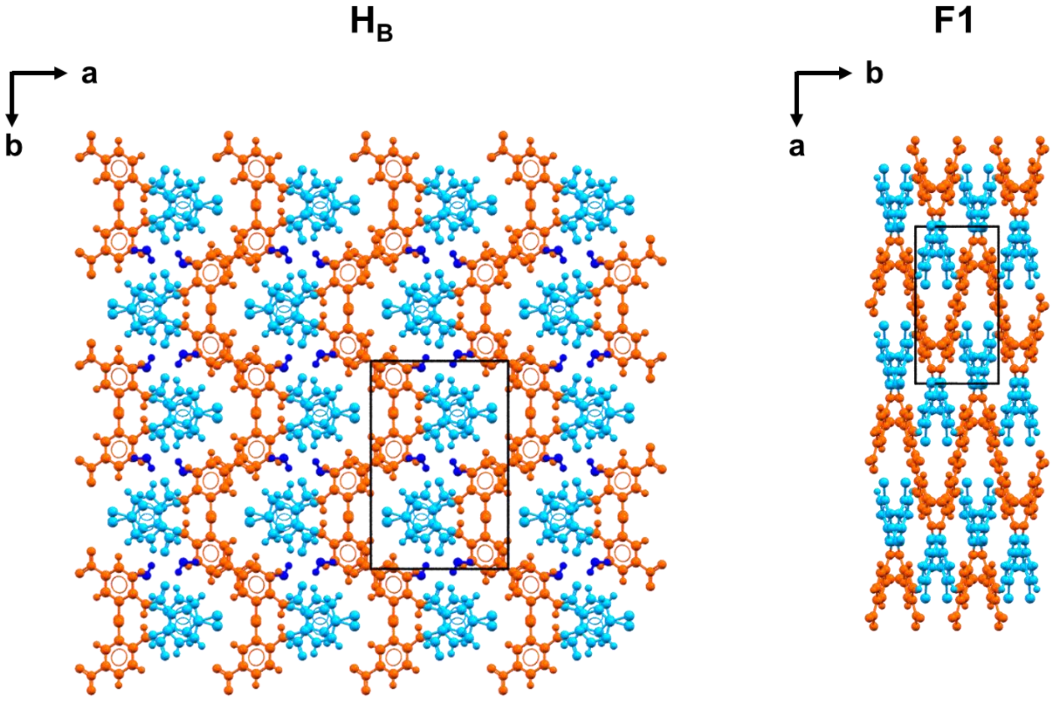

Figure S14. Schematic of packing in H_B and F1. Each NCL molecule is colored so that the nitrophenyl ring is orange and the phenol ring is light blue. Water molecules in H_B are dark blue.
